# Introductory Lecture

**Published:** 1872-10

**Authors:** 


INTRODUCTORY LECTURE.—The lecture introductory to the regular course of lectures, in the Buffalo Medical College will be given by Prof. Rochester, Wednesday evening Nov. 6th in the college Amphitheatre, commencing at 7½ o'clock. Members of the profession are invited to be present. The subjects usually chosen on these occasions are of interest, and those who attend never fail of an instructive and pleasant entertainment.

